# What Unrelated Hematopoietic Stem Cell Transplantation in Thalassemia Taught us about Transplant Immunogenetics

**DOI:** 10.4084/MJHID.2016.048

**Published:** 2016-10-20

**Authors:** Giorgio La Nasa, Adriana Vacca, Roberto Littera, Eugenia Piras, Sandro Orru, Marianna Greco, Carlo Carcassi, Giovanni Caocci

**Affiliations:** 1 Bone Marrow Transplant Center, R. Binaghi Hospital - ASL 8, Cagliari, Italy; 2 Hematology Unit, Department of Medical Sciences, University of Cagliari, Italy; 3 Regional Transplant Center, R. Binaghi Hospital - ASL 8, Cagliari, Italy; 4 Medical Genetics, Department of Medical Sciences, University of Cagliari, Italy

## Abstract

Although the past few decades have shown an improvement in the survival and complication-free survival rates in patients with beta-thalassemia major and gene therapy is already at an advanced stage of experimentation, hematopoietic stem cell transplantation (HSCT) continues to be the only effective and realistic approach to the cure of this chronic non-malignant disease. Historically, human leukocyte antigen (HLA)-matched siblings have been the preferred source of donor cells owing to superior outcomes compared with HSCT from other sources. Nowadays, the availability of an international network of voluntary stem cell donor registries and cord blood banks has significantly increased the odds of finding a suitable HLA matched donor. Stringent immunogenetic criteria for donor selection have made it possible to achieve overall survival (OS) and thalassemia-free survival (TFS) rates comparable to those of sibling transplants. However, acute and chronic graft-versus-host disease (GVHD) remains the most important complication in unrelated HSCT in thalassemia, leading to significant rates of morbidity and mortality for a chronic non-malignant disease. A careful immunogenetic assessment of donors and recipients makes it possible to individualize appropriate strategies for its prevention and management. This review provides an overview of recent insights about immunogenetic factors involved in GVHD, which seem to have a potential role in the outcome of transplantation for thalassemia.

## Unrelated Hematopoietic Stem Cell Transplantation for Thalassemia

Beta thalassemia major is an inherited hemoglobinopathy characterized by anemia and chronic hemolysis,^1^ affecting several dimensions of the patient’s life such as education, free-time, physical activities, skills, family life and often results in anxiety, depression and lower health related quality of life (HRQoL).^2^,^3^ Blood transfusions and chelation practice have been routinely performed in the management of these patients.^2^,^4^,^5^

Until now, despite the promising outcomes coming from clinical trials on gene therapy, hematopoietic stem cell transplantation (HSCT) remains the only curative approach for this severe genetic disorder. Since its first successful application in 1981, more than 3,000 thalassemia patients have been transplanted worldwide. The 5-year probabilities of overall survival (OS) and thalassemia free survival (TFS) are currently estimated to be between 87% to 97% and 80% to 89% respectively.^6^ Despite successful HSCT eliminates the need for blood transfusions, pre-transplant clinical conditions, as well as clinical complications and adverse effects of therapy, contribute to the impairment of HRQoL.^7^ Nevertheless, a study in a large cohort of ex-thalassemia patients who underwent HSCT more than 20 years ago showed that the ex-patients, their sibling donors, and the general population had a very similar HRQoL and that the latter was even better in the ex-patients than a control group of thalassemic patients treated conventionally with blood transfusions and iron chelation therapy.^8^

Human leukocyte antigen (HLA) compatible marrow stem cells from identical sibling donor represent the goal choice for thalassemia patients requiring allogeneic HSCT. However, the probability of finding a compatible donor within the family ranges from 25% to 30%. For the remaining patients, HSCT from an unrelated voluntary donor is a feasible alternative. In recent years, the growing availability of alternative stem cell sources (marrow from an unrelated or haploidentical donor and hematopoietic stem cells from umbilical cord blood) has made HSCT a feasible option for an increasing number of patients in search of a compatible donor. By applying highly stringent criteria for donor selection, the outcome of unrelated HSCT is not much different from that obtained when using a compatible sibling with a 5-year probability of OS and TFS of 84% and 75%, respectively.^9^ So far, more than 300 thalassemia patients have been transplanted from an unrelated donor. Table 1 illustrates the outcome of unrelated HSCT and cord blood transplantation (CBT) performed to cure children and young adults with beta thalassemia major, using both myelo-ablative and, more recently, reduced-intensity conditioning regimens.

## Graft Versus Host Disease in Unrelated HSCT

Unfortunately, acute and chronic Graft Versus Host Disease (GvHD), a potentially multi-systemic disorder caused by immunoeffector donor lymphocytes that destroy host tissues, remains the main treatment-related cause of death in unrelated HSCT in thalassemia.^10^ The prevention of acute or chronic GvHD in thalassemia patients undergoing unrelated HSCT represents a critical issue. GvHD is actually a complication without any benefits (Graft-versus-Leukemia effect) for patients with a chronic non-malignant disease. Therefore, the minimization of GvHD immunogenetic risk is mandatory. Indeed, GvHD is not only associated with a high mortality risk, but it can also seriously impair the post-transplant HRQoL. Although immunosuppressive therapy represents the fundamental approach to the prevention of this severe complication, careful donor selection can result in marked improvement in the outcomes. The incidence of GvHD is highly variable from individual to individual due to several modifiable and non-modifiable risk factors, such us the conditioning regimen intensity, the stem cell source, the degree of human leukocyte antigen (HLA) mismatch, the previous alloimmunization in the donor, the use of total body irradiation, the GvHD prophylaxis, the severity of individual organ insufficiencies, the female *vs* male donor recipient, the recipient age and the transplantation risk class according to the Pesaro classification (hepatomegaly, liver fibrosis and inadequate chelation therapy).^11^

Historically, the first series of 32 transplants from bone marrow HSC of unrelated donor (median age 14 years, range 2–28 years) was published in 2002.^12^ Severe acute GvHD was registered in 41% of cases and chronic GvHD in 25%. In 2005, the same research group extended the study to 68 thalassemia patients.^13^ In this cohort, 25% of patients received anti-thymocyte globulin (ATG, 3.5 mg/kg) for GvHD prophylaxis, in addition to cyclosporine (CSP) and short courses of methotrexate (MTX). Nevertheless, 40% of the patients developed severe acute GVHD and 18% chronic GVHD. In the following years, the Italian cohort of thalassemia patients transplanted from unrelated donors rose to over 100 cases; in 2011, a report at the annual ASH meeting, presented outcomes of 122 thalassemia patients (median age 10,5 range 1–35 years).^9^ Interestingly, the myeloablative conditioning regimen in 40 patients was slightly different, since it was based on the combination of conventional drugs with treosulfan (Treo).^14^ All patients received homogeneous GvHD prophylaxis consisting of CSP, short courses MTX and ATG, and donor selection was based on high resolution HLA typing. Acute and chronic GvHD dropped to 28% and 13%, respectively. This treosulfan-based preparation proved to be safe and effective for thalassemia patients undergoing allogeneic HSCT.

With regard to alternative stem cell sources, an experience with granulocyte colony-stimulating factor (G-CSF)-mobilized peripheral HSC was described in 2012.^15^ GvHD prophylaxis consisted of CSP, mycophenolate mofetil (MMF), short courses MTX and ATG. The mean dose of CD34+ cells that were infused was 6.81×10^6^/kg. These authors reported a very low percentage of acute GvHD (9.6%) and no cases of chronic GvHD. These findings are even more surprising if we consider that the peripheral source of HSC is usually related to a higher rate of GvHD if compared to bone marrow HSC. Current research is focused on the use of cord blood (CB) as an alternative HSC source in unrelated transplantation for thalassemia. In a pediatric setting, this option has the advantage of being immediately available when required and CB is not as difficult to match as stem cells from an adult volunteer donor. Incidence of GvHD is widely variable both for acute (23–47%) and chronic form (11–35%).^16^,^17^,^18^ Unfortunately, rejection rates remain very high (Table 1).^17^,^18^

Overall, GvHD causes severe morbidity, and long survivors have a severely compromised HRQoL. Recently, there has been a growing interest in immunogenetic variables that seem to have a potential role in the outcome of transplantation for thalassemia, capable of predicting the onset of GvHD

## Role of HLA-DPB1

**Figure 1:**
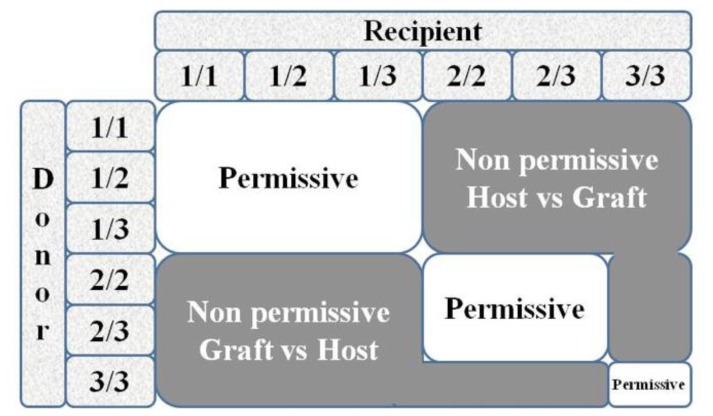
Risk assessment of acute graft-versus-host disease and rejection according to permissive and nonpermissive HLA-DPB1 mismatches in donor and recipient pairs.

Over the past years, the concept of “permissive” or “non-permissive” HLA-DPB1 disparities and their influence on transplantation outcome, with specific reference to acute GvHD and rejection, has aroused considerable interest. HLA-DP antigens are involved in both humoral and cellular alloresponse and are encoded by genes at the DPA1 locus, which displays limited polymorphism, at the highly polymorphic DPB1 locus.^19^ Because of the weak linkage disequilibrium existing between the DR/DQ and DP loci, only 20% of matched unrelated donor-recipient pairs are also compatible for HLA-DPB1, while about 80% are transplanted across the HLA-DPB1 barrier. A previous study has proposed an algorithm based on in vitro donor/recipient immune-reactivity, which was found to be associated with a significantly increased risk of transplantation-related mortality (TRM) and severe acute GVhD in patients undergoing transplantation for malignant hematopoietic disorders (Figure 1).^20^ The algorithm divided HLA-DPB1 alleles into 3 groups according to in vitro immunogenicity testing and classified the risk of rejection or GvHD for each of the 6 different combinations present in diploid cells. On the basis of cross-reactive patterns by alloreactive T cells involved in HSCT rejection targeted to HLADPB1*0901, the algorithm divides HLA-DPB1 alleles into 3 groups with high (HLA-DPB1*0901,*1001,*1701), intermediate (HLA-DPB1 *0301,*1401,*4501), or low (most other HLADPB1 alleles) immunogenicity, on the basis of a shared alloreactive T-cell epitope.^20^

A study on 72 thalassemia patients receiving unrelated HSCT confirmed that the risk of rejection was associated with the presence of non-permissive HLA-DPB1 mismatches in the host-versus-graft (HvG) direction, as predicted by the algorithm.^21^ TFS was reduced in patients with non-permissive HLA-DPB1 mismatches in HvG (59%) or GvHD (60%) direction, as compared to the matched or permissive group (78%). The authors suggested that implementation of standard criteria for donor selection may highlight the crucial role of immunogenetic factors in the occurrence of complications due to alloreactivity following unrelated donor HSCT for beta-thalassemia. The clinical predictive value of the aforesaid algorithm was retrospectively confirmed in a study of 621 unrelated HSCT from the Italian Bone Marrow Donor Registry and in a large cohort of 8,539 transplanted patients.^22^,^23^ Non-permissive mismatches were associated with a significantly increased risk of TRM and severe acute GvHD, compared to permissive mismatches. These results suggest that HSCT from unrelated donors with a non-permissive T-cell-epitope mismatch at HLA-DPB1 deserves careful consideration, particularly in the setting of a chronic non malignant disease like thalassemia, where it is important to lower the risks of GvHD and TRM. The DPB1 algorithm for donor selection is a powerful tool for the prediction of complications in thalassemia patients undergoing unrelated HSCT.

## Role of HLA-G

Another molecule that could play a role in immune modulation after HSCT is human leukocyte antigen G (HLA-G). HLA-G is a non-classical MHC class I molecule of low polymorphism and restricted tissue distribution. It is highly expressed in trophoblast cells and plays a critical role in embryo implantation and pregnancy by contributing to maternal immune tolerance of the fetus through its immunosuppressive effects on all types of immune cells.^24^ The HLA-G gene is located on the short arm of chromosome 6, in the HLA region 6p21.2–21.3, between the HLA-A and HLA-F genes.^25^ The HLA-G primary transcript generates 7 alternative messenger ribonucleic acids (mRNAs) that encode 3 different soluble isoforms sHLA-G1 or HLA-G5, HLA-G6, and HLA-G7 and 4 isoforms with transmembrane and cytoplasmic domains HLA-G1, -G2, -G3, -G4.^26^ HLA-G allelic variants may also be characterized by a 14-basepair (bp) deletion-insertion polymorphism located in the 3′-untranslated region of the HLA-G gene. The presence of the 14-bp insertion is known to generate additional mRNA splicing.^26^,^27^ It would seem that spliced mRNAs are more stable than their corresponding cDNA transcript counterparts, and thus determine an increment in HLA-G expression. The influence of the 14-bp deletion/insertion polymorphism on the outcome of HSCT was retrospectively investigated in 53 patients affected by thalassemia major transplanted from an unrelated donor.^28^ The risk of developing acute GvHD was higher in patients homozygous for the 14-bp deletion compared to patients carrying the 14-bp insertion. The authors suggested that this finding could be related to the increased production of HLA-G molecules and proposed the evaluation of the HLA-G 14-bp insertion-deletion polymorphism in the pre-transplantation risk-assessment process.

## Role of CTLA-4

The co-receptor cytotoxic T-lymphocyte antigen-4 (CTLA-4) is a molecule involved in regulating the duration and severity of immune response after HSCT. CTLA-4, also known as CD152, is one of the most important members of the immunoglobulin superfamily and it is a vital restraining regulator of T-cell proliferation and activation, inducing Fas-independent apoptosis of activated T cells.^29^,^30^ CTLA-4 is akin to CD28 molecule both of them bind B7.1 and B7.2 on antigen-presenting cells (APC), with a much higher affinity of CTLA-4 than CD28. The mechanisms through which CTLA-4 plays a role as a negative regulator are multiple, including the reduction of both interleukin (IL)-2 and IL-2 receptor production and the T cells block at the G1 phase in the cell cycle.^31^,^32^ The CTLA4 gene is located on human chromosome 2q33 and consists of four exons: the exon 1 contains the leader peptide sequence, the exon 2 the ligand binding site, the exon 3 encodes the transmembrane region, and the exon 4 the cytoplasmic tail. Differential splicing of the CTLA-4 transcript results in a full length transmembrane form (exons 1–4), soluble CTLA-4 (lacking exon 3), and a transcript encoding only for exons 1 and 4;^33^ the alternative splicing that generates these isoforms seems to be regulated by genetic polymorphisms.^34^ Polymorphisms of the CTLA-4 gene have been associated with autoimmune diseases and, more recently, with the outcome of allogeneic HSCT in leukemia patients.^35^ A previous study reported the role of CTLA-4 gene polymorphisms in the onset of acute GvHD following unrelated HSCT in a cohort of thalassemia patients transplanted at a mean age of 13 years.^36^ The genotype distribution for each CTLA-4 polymorphism was compared according to the presence or absence of acute GvHD. Patients homozygous for the CT60-A allele had a significantly higher probability of developing acute GvHD than patients with the CT60-G allele. According to the authors, the protective effect of the G-allele may be due to down-regulation of soluble CTLA-4 expression levels. On the other hand, the higher levels of soluble CTLA-4 associated with CT60-A homozygosity may hamper binding of B7 with membrane-bound CTLA-4 expressed on activated T cells and thus increase their reactivity. These data indicate that the genetic risk factors for GVHD may also depend on the recipient genotype and that the portion of residual T-cells may play a major role in the outcome of HSCT for thalassemia.

## Role of Killer Cell Immunoglobulin-like Receptors (KIRs)

HLA class I molecules and killer cell immunoglobulin-like receptors (KIRs) are of pivotal importance for regulating natural killer (NK) cell function. Previous studies have shown that donor NK cell alloreactivity exerts a graft-versus-leukemia effect which reduces the risk of GvHD through lysis of recipient antigen-presenting cells (APCs).^37^

In humans, two different types of HLA class-I-specific inhibitory receptors exist: KIRs, also referred to as CD158, that in most instances, recognize the polymorphic HLA-A, -B, and -C molecules, and CD94/NKG2A (CD94/CD159a), a heterodimer related to C-type lectins that recognize HLA-E, a non-classical MHC molecule characterized by a limited polymorphism. KIRs are transmembrane glycoproteins containing two (D1 and D2, designated KIR2D) or three (D0, D1, and D2, designated KIR3D) extracellular C2-type Ig-like domains, while KIR2D receptors bind HLA-C alleles, KIR3D receptors bind HLA-A and HLA-B alleles.^38^,^39^ Depending on to the function performed, the intracytoplasmatic domains of KIRs can feature either a short (called activating KIRs) or a long (called inhibitory KIRs) cytoplasmic tail, “S” or “L” in the nomenclature, respectively.^40^ In particular, reduced activity of NK cells may be related to the balancement between inhibitory (KIR2DL1, KIR2DL2, KIR2DL3, KIR2DL4, KIR2DL5, KIR3DL1, KIR3DL2, KIR3DL3) and activating (KIR2DS1, KIR2DS2, KIR2DS3, KIR3DS1) KIR genes.^41^ All people may differ strongly in the activating KIR content. In particular, two different types of KIR haplotypes can be distinguished in humans: A and B. In general, haplotypes A are beneficial in NK responses to pathogens, whereas haplotypes B are associated with low frequencies of pregnancy disorders.^42^ KIR haplotype A contains six inhibitory KIR genes and a single activating KIR gene (KIR2DS4), while KIR haplotype B contains various combinations of both activating and inhibitory KIR genes.^43^,^44^ Moreover, KIR hapotype A may completely lack expression of activating KIRs on the NK cell surface depending on functional or non-functional variants of the only activating receptor KIR2DS4.^45^ These receptors prevent NK cell-mediated attack against normal autologous cells and allow the killing of cells that upon tumor transformation or viral infection present compromised HLA class-I expression (“missing self hypothesis”).^46^

**Figure 2:**
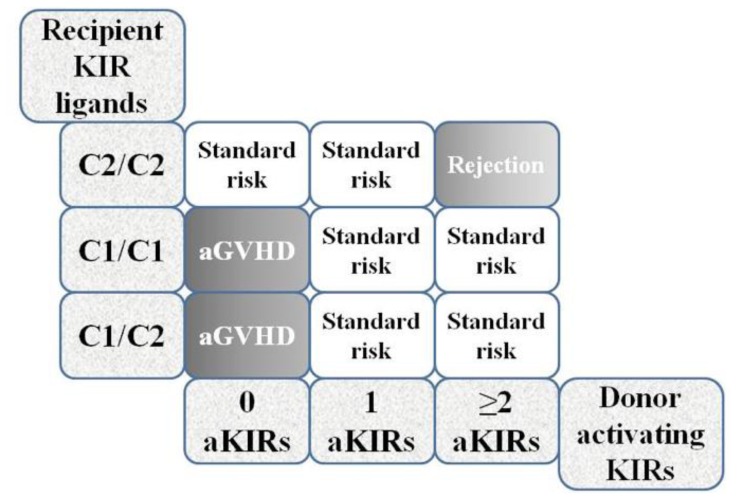
Algorithm showing the risk of developing acute GvHD or rejection after unrelated HSCT according to recipient KIR ligands and the number of donors activating KIRs. aGVHD= acute Graft Versus Host Disease.

Until now, few studies have reported the influence of KIRs on HSCT. A study investigating the impact of KIRs and their ligands on the outcome of unrelated HSCT was conducted in a cohort of transfusion-dependent beta-thalassemia patients.^47^,^48^ Donors and recipients were divided into two groups according to combinations of group A and group B KIR haplotypes: those homozygous for KIR haplotype A (AA) and those either heterozygous or homozygous for KIR haplotype B (AB+BB). The authors found that patients transplanted from AA homozygotes had a fourfold risk of developing moderate acute GvHD and a sevenfold risk of developing severe acute GvHD. Considering that the A haplotype only includes one stimulatory KIR (2DS4), which in most cases is not expressed on the NK cell membrane and thus remains ineffective, the authors postulated that NK cells in donors expressing only the A haplotype could display a lower efficiency in killing recipient APCs. By interpolating the number of donor activating KIRs with recipient HLA-C KIR ligands, the authors proposed an algorithm that is apparently capable of stratifying patients according to the immunogenetic risk of complications following transplantation (Figure 2).^49^

Based on the amino acid sequence at position 80, HLA-C allotypes can be divided into two groups. HLA-C1 has an asparagine at position 80 whereas HLA-C2 has a lysine. According to reports in the literature, HLA-C1 binds the KIR2DL2 and KIR2DL3 inhibitory receptors and the KIR2DS2 and KIR2DS3 activating receptors, whereas HLA-C2 binds the KIR2DL1 inhibitory receptor and the KIR2DS1 activating receptor. Homozygosity or heterozygosity for C1 and C2 KIR ligand groups and the number of donors activating KIR genes have been found associated with HSCT outcome. Donors homozygous for KIR haplotype A who only carry deletion variants of KIR2DS4 (the only activating KIR of the A haplotype) seem to be incapable of expressing functional activating KIRs on the NK cell surface. The purpose of the algorithm was to assess the risk of developing acute GvHD or rejection according to combinations of recipient HLA KIR ligand groups C1/C2, C1/C1 and C2/C2 and the number of donors activating KIRs (0, 1, ≥2). The highest risk of acute GvHD was observed for C1/C2 heterozygotes transplanted from donors completely lacking functional activating KIRs. Conversely, the risk of acute GvHD was entirely absent in C2/C2 homozygotes transplanted from donors possessing ≥2 activating KIRs. Consistent with these findings, the authors proposed using the algorithm as a supportive tool in pre-HSCT risk assessment.^49^

## Conclusions

**Figure 3:**
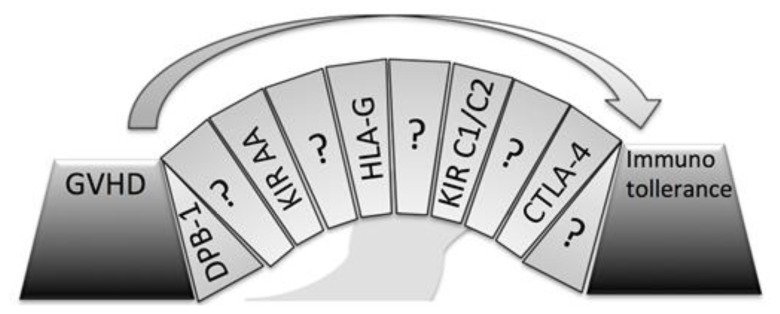
The path from GvHD to immune tolerance is like a bridge where many variables are still missing.

More in-depth comprehension of immunogenetic factors associated with a reduced risk of GvHD and TRM may possibly assist us in the selection of an ideal donor for a patient affected by thalassemia. Understanding the role of genetic factors capable of influencing the onset of GvHD is fundamental to the increasingly safer application of unrelated HSCT in thalassemia, particularly if we consider that GvHD is the leading cause of morbidity and mortality in this setting.^8^ Several variables seem to have a role in GvHD onset, but a clear vision of the whole problem is still missing (Figure 3).

Careful assessment of all the immunogenetic variables discussed in this review is needed to optimize the accuracy of pre-transplantation risk assessment. Data on HLA-G, CTLA-4 and KIR variables need further validations within prospective studies and multivariate analyses before a standard use in clinical practice could be suggested. Such an approach, with the support of mathematical software and statistics, should form a sound informative basis from which to derive strategies for the prevention of acute GvHD following HSCT for a chronic non-malignant disease.^50^ In conclusion, unrelated HSCT outcomes in thalassemia will continue to improve, with lower TRM and better OS and TFS rates. Prevention of GvHD through careful evaluation of immunogenetic features of donor and recipient pairs has a key role in this curative approach to beta thalassemia major.

## Key Issues

- Allogeneic hematopoietic stem cell transplantation (HSCT) is currently the only treatment capable of definitively correcting the defective erythropoiesis in beta thalassemia major.
- The probability of finding a compatible donor within the family ranges from 25 to 30%. For other patients, HSCT from an unrelated voluntary donor is a feasible alternative.
- By applying highly stringent criteria for donor selection, the outcome of unrelated HSCT is not much different from that obtained when using a compatible sibling
- Graft versus host disease (GvHD) remains a significant treatment-related cause of death in unrelated HSCT for thalassemia
- Human leukocyte antigen-DPB1 (HLA-DPB1) disparities between donors and recipients represent a powerful tool for the prediction of GvHD in unrelated HSCT.
- Human leukocyte antigen G (HLA-G) and cytotoxic T lymphocyte associated antigen-4 (CTLA-4) could play a role in immune modulation after HSCT
- Killer immunoglobulin-like receptors (KIRs) have a prominent role in the development of GVHD

## Figures and Tables

**Table 1:** Clinical outcomes of unrelated hematopoietic stem cell transplantation and cord blood transplantation in young adult and pediatric thalassemia patients.

First author	N° pts	Source	Age median years (range)	OS (%)	TFS (%)	TRM (%)	Rejection (%)	aGvHD (%)	cGvHD (%)
**La Nasa (2002)12**	32	BM	14 (2–28)	79	66	19	12.5	41	25
**La Nasa (2005)13**	68	BM	15 (2–37)	79.3	65.8	20	13	40	18
**Hongeng (2006)51**	21	BM	4 (0.7–12)	85.7	71	14.3	14.3	42	14
**Locatelli (2011)9**	122	BM	10.5 (1–35)	84	75	16.4	13.1	28	13
**Ruggeri (2011)17**	35	CB	4 (0.5–14)	62	21	34	57	23	16
**Jaing (2012)16**	35	CB	5.5 (1.2–14)	88.5	88.5	11.4	0	47	35
**Li (2012)15**	52	BM/PB	6 (2–15)	92.3	90.4	7.7	1.9	9.6	0
**Anurathapan (2014)52**	26	BM/PB	8 (2–10)	94	82	7	0	28	15
**Shah (2015)18**	9	CB	3.8 (1.5–7)	100	56%	0	44%	33%	11%

BM: bone marrow; CB: cord blood; PB: peripheral blood; OS: overall Survival; TFS: Thalassemia Free Survival; TRM: Transplantation-related mortality; aGvHD: acute graft versus host disease; cGvHD: chronic graft versus host disease.

## References

[1] Rund D (2016). Thalassemia 2016: Modern medicine battles an ancient disease. Am J Hematol.

[2] Borgna-Pignatti C (2010). The life of patients with thalassemia major. Haematologica.

[3] Caocci G, Efficace F, Ciotti F, Roncarolo MG, Vacca A, Piras E (2012). Health related quality of life in Middle Eastern children with beta-thalassemia. BMC Blood Disord.

[4] Modell B, Khan M, Darlison M, Westwood MA, Ingram D, Pennell DJ (2008). Improved survival of thalassaemia major in the UK and relation to T2* cardiovascular magnetic resonance. J Cardiovasc Magn Reson.

[5] Borgna-Pignatti C, Marsella M (2015). Iron Chelation in Thalassemia Major. Clin Ther.

[6] Mathews V, Srivastava A, Chandy M (2014). Allogeneic stem cell transplantation for thalassemia major. Hematol Oncol Clin North Am.

[7] Caocci G, Efficace F, Ciotti F, Roncarolo MG, Vacca A, Piras E (2011). Prospective assessment of health-related quality of life in pediatric patients with beta-thalassemia following hematopoietic stem cell transplantation. Biol Blood Marrow Transplant.

[8] La Nasa G, Caocci G, Efficace F, Dessì C, Vacca A, Piras E (2013). Long-term health-related quality of life evaluated more than 20 years after hematopoietic stem cell transplantation for thalassemia. Blood.

[9] Locatelli F, Littera R, Pagliara D (2011). Outcome of unrelated donor bone marrow transplantation for thalassemia major patients. Blood.

[10] Pasquini MC, Griffith LM, Arnold DL, Atkins HL, Bowen JD, Chen JT (2010). Hematopoietic stem cell transplantation for multiple sclerosis: collaboration of the CIBMTR and EBMT to facilitate international clinical studies. Biol Blood Marrow Transplant.

[11] Lucarelli G, Andreani M, Angelucci E (2002). The cure of thalassemia by bone marrow transplantation. Blood Rev.

[12] La Nasa G, Giardini C, Argiolu F, Locatelli F, Arras M, De Stefano P (2002). Unrelated donor bone marrow transplantation for thalassemia: the effect of extended haplotypes. Blood.

[13] La Nasa G, Argiolu F, Giardini C, Pession A, Fagioli F, Caocci G (2005). Unrelated bone marrow transplantation for beta-thalassemia patients: The experience of the Italian Bone Marrow Transplant Group. Ann N Y Acad Sci.

[14] Bernardo ME, Piras E, Vacca A, Giorgiani G, Zecca M, Bertaina A (2012). Allogeneic hematopoietic stem cell transplantation in thalassemia major: results of a reduced-toxicity conditioning regimen based on the use of treosulfan. Blood.

[15] Li C, Wu X, Feng X, He Y, Liu H, Pei F (2012). A novel conditioning regimen improves outcomes in β-thalassemia major patients using unrelated donor peripheral blood stem cell transplantation. Blood.

[16] Jaing TH, Hung IJ, Yang CP, Chen SH, Chung HT, Tsay PK, Wen YC (2012). Unrelated cord blood transplantation for thalassaemia: a single-institution experience of 35 patients. Bone Marrow Transplant.

[17] Ruggeri A, Eapen M, Scaravadou A, Cairo MS, Bhatia M, Kurtzberg J (2011). Umbilical cord blood transplantation for children with thalassemia and sickle cell disease. Biol Blood Marrow Transplant.

[18] Shah SA, Shah KM, Patel KA, Anand AS, Talati SS, Panchal HP (2015). Unrelated Umbilical Cord Blood Transplant for Children with β-Thalassemia Major. Indian J Hematol Blood Transfus.

[19] Marsh SG (2016). Nomenclature for factors of the HLA system, update February 2016. Hum Immunol.

[20] Zino E, Frumento G, Marktel S, Sormani MP, Ficara F, Di Terlizzi S (2004). A T-cell epitope encoded by a subset of HLA-DPB1 alleles determines nonpermissive mismatches for hematologic stem cell transplantation. Blood.

[21] Fleischhauer K, Locatelli F, Zecca M, Orofino MG, Giardini C, De Stefano P (2006). Graft rejection after unrelated donor hematopoietic stem cell transplantation for thalassemia is associated with nonpermissive HLA-DPB1 disparity in host-versus-graft direction. Blood.

[22] Crocchiolo R, Zino E, Vago L, Oneto R, Bruno B, Pollichieni S, Gruppo Italiano Trapianto di Midollo Osseo, Cellule Staminale Ematopoietiche (CSE) e Terapia Cellulare; Italian Bone Marrow Donor Registry (2009). Nonpermissive HLA-DPB1 disparity is a significant independent risk factor for mortality after unrelated hematopoietic stem cell transplantation. Blood.

[23] Fleischhauer K, Shaw BE, Gooley T, Malkki M, Bardy P, Bignon JD (2012). Effect of T-cell-epitope matching at HLA-DPB1 in recipients of unrelated-donor haemopoietic-cell transplantation: a retrospective study. Lancet Oncol.

[24] Kuroki K, Maenaka K (2007). Immune modulation of HLA-G dimer in maternal-fetal interface. Eur J Immunol.

[25] Koller BH, Geraghty DE, DeMars R, Duvick L, Rich SS, Orr HT (1989). Chromosomal organization of the human major histocompatibility complex class I gene family. J Exp Med.

[26] Donadi EA, Castelli EC, Arnaiz-Villena A, Roger M, Rey D, Moreau P (2011). Implications of the polymorphism of HLA-G on its function, regulation, evolution and disease association. Cell Mol Life Sci.

[27] Rebmann V, da Silva Nardi F, Wagner B, Horn PA (2014). HLA-G as a tolerogenic molecule in transplantation and pregnancy. J Immunol Res.

[28] La Nasa G, Littera R, Locatelli F, Lai S, Alba F, Caocci G (2007). The human leucocyte antigen-G 14-basepair polymorphism correlates with graft-versus-host disease in unrelated bone marrow transplantation for thalassaemia. Br J Haematol.

[29] Sun T, Zhou Y, Yang M, Hu Z, Tan W, Han X (2008). Functional genetic variations in cytotoxic T-lymphocyte antigen 4 and susceptibility to multiple types of cancer. Cancer Res.

[30] Scheipers P, Reiser H (1998). Fas-independent death of activated CD4(+) T lymphocytes induced by CTLA-4 crosslinking. Proc Natl Acad Sci U S A.

[31] Sharpe AH, Freeman GJ (2002). The B7–28 superfamily. Nat Rev Immunol.

[32] Alegre ML, Frauwirth KA, Thompson CB (2001). T-cell regulation by CD28 and CTLA-4. Nat Rev Immunol.

[33] Appleman LJ, Berezovskaya A, Grass I, Boussiotis VA (2000). CD28 costimulation mediates T cell expansion via IL-2-independent and IL-2-dependent regulation of cell cycle progression. J Immunol.

[34] Ueda H, Howson JM, Esposito L, Heward J, Snook H, Chamberlain G (2003). Association of the T-cell regulatory gene CTLA4 with susceptibility to autoimmune disease. Nature.

[35] Pérez-García A, De la Cámara R, Román-Gómez J, Jiménez-Velasco A, Encuentra M, Nieto JB, GVHD/Immunotherapy Committee of the Spanish Group of Hematopoietic Stem Cell Transplantation (2007). CTLA-4 polymorphisms and clinical outcome after allogeneic stem cell transplantation from HLA-identical sibling donors. Blood.

[36] Orrù S, Orrù N, Manolakos E, Littera R, Caocci G, Giorgiani G (2012). Recipient CTLA-4*CT60-AA genotype is a prognostic factor for acute graft-versus-host disease in hematopoietic stem cell transplantation for thalassemia. Hum Immunol.

[37] Ruggeri L, Capanni M, Urbani E, Perruccio K, Shlomchik WD, Tosti A (2002). Effectiveness of donor natural killer cell alloreactivity in mismatched hematopoietic transplants. Science.

[38] Natarajan K, Dimasi N, Wang J, Mariuzza RA, Margulies DH (2002). Structure and function of natural killer cell receptors: multiple molecular solutions to self, nonself discrimination. Annu Rev Immunol.

[39] Joyce MG, Sun PD (2011). The structural basis of ligand recognition by natural killer cell receptors. J Biomed Biotechnol.

[40] Moretta A, Bottino C, Vitale M, Pende D, Biassoni R, Mingari MC (1996). Receptors for HLA class-I molecules in human natural killer cells. Annu Rev Immunol.

[41] Bashirova AA, Martin MP, McVicar DW, Carrington M (2006). The killer immunoglobulin-like receptor gene cluster: tuning the genome for defense. Annu Rev Genomics Hum Genet.

[42] Parham P, Norman PJ, Abi-Rached L, Guethlein LA (2012). Human-specific evolution of killer cell immunoglobulin-like receptor recognition of major histocompatibility complex class I molecules. Philos Trans R Soc Lond B Biol Sci.

[43] Hsu KC, Chida S, Geraghty DE, Dupont B (2002). The killer cell immunoglobulin-like receptor (KIR) genomic region: gene-order, haplotypes and allelic polymorphism. Immunol Rev.

[44] Bontadini A, Testi M, Cuccia MC, Martinetti M, Carcassi C, Chiesa A (2006). Distribution of killer cell immunoglobulin-like receptors genes in the Italian Caucasian population. J Transl Med.

[45] Middleton D, Gonzelez F (2010). The extensive polymorphism of KIR genes. Immunology.

[46] Kärre K (2008). Natural killer cell recognition of missing self. Nat Immunol.

[47] La Nasa G, Littera R, Locatelli F, Giardini C, Ventrella A, Mulargia M (2007). Status of donor-recipient HLA class I ligands and not the KIR genotype is predictive for the outcome of unrelated hematopoietic stem cell transplantation in beta-thalassemia patients. Biol Blood Marrow Transplant.

[48] Littera R, Orrù N, Vacca A, Bertaina A, Caocci G, Mulargia M (2010). The role of killer immunoglobulin-like receptor haplotypes on the outcome of unrelated donor haematopoietic SCT for thalassaemia. Bone Marrow Transplant.

[49] Littera R, Orrù N, Caocci G, Sanna M, Mulargia M, Piras E (2012). Interactions between killer immunoglobulin-like receptors and their human leucocyte antigen Class I ligands influence the outcome of unrelated haematopoietic stem cell transplantation for thalassaemia: a novel predictive algorithm. Br J Haematol.

[50] Caocci G, Baccoli R, Vacca A, Mastronuzzi A, Bertaina A, Piras E (2010). Comparison between an artificial neural network and logistic regression in predicting acute graft-vs-host disease after unrelated donor hematopoietic stem cell transplantation in thalassemia patients. Exp Hematol.

[51] Hongeng S, Pakakasama S, Chuansumrit A, Sirachainan N, Kitpoka P, Udomsubpayakul U (2006). Outcomes of transplantation with related- and unrelated-donor stem cells in children with severe thalassemia. Biol Blood Marrow Transplant.

[52] Anurathapan U, Pakakasama S, Mekjaruskul P, Sirachainan N, Songdej D, Chuansumrit A (2014). Outcomes of thalassemia patients undergoing hematopoietic stem cell transplantation by using a standard myeloablative versus a novel reduced-toxicity conditioning regimen according to a new risk stratification. Biol Blood Marrow Transplant.

